# The “Information Paradox” in crime prevention: how spatial context and age moderate the impact of safety advisories on residents’ perception of safety

**DOI:** 10.3389/fpubh.2026.1868817

**Published:** 2026-08-28

**Authors:** Zengli Wang

**Affiliations:** 1 Department of Remote Sensing, College of Geography and Remote Sensing, Hohai University, Nanjing, China; 2 Jiangsu Key Laboratory of Soil and Water Processes in Watershed, College of Geography and Remote Sensing, Hohai University, Nanjing, China

**Keywords:** age interaction, crime prevention, Information Paradox, perception of safety, spatial context, urban public health

## Abstract

**Background:**

Effective crime prevention communication is a cornerstone of urban public health and psychological well-being. However, the psychological impact of safety advisories on residents remains poorly understood. This study investigates the “Information Paradox,” the phenomenon where crime prevention tips may inadvertently heighten fear rather than provide reassurance, and explores how spatial delivery channels and demographic factors moderate this relationship.

**Methods:**

Drawing on a survey of residents in Nanjing, China, this study employed ordinal logistic regression (OLR) models to analyze the influence of multi-channel information (e.g., Community Noticeboards, unit entrances, elevator lobbies, and digital platforms) on perceived safety. Interaction terms were introduced to examine the moderating role of age cohorts, shifting the focus from technological access to varying risk sensitivities and socio-economic responsibilities across life stages.

**Results:**

The results reveal a significant spatial divergence in information perception. Crime prevention tips at unit entrances significantly enhance safety perceptions (*B* = 0.370, *p* < 0.05), acting as signs of guardianship. Conversely, reminders in elevator lobbies correspond to a sharp decline in security feelings (*B* = −0.741, *p* < 0.01) triggering a micro-spatial warning effect where structural entrapment overrides message intent. Furthermore, interaction analysis shows that the early-to-middle-aged groups exhibit a systematically lower likelihood of high safety alignment. Notably, the interaction terms for (31–40 year) × Television News (*B* = −1.985, *p* = 0.002, OR = 0.137) and (41–50 years) × Mobile Applications (*B* = −1.556, *p* = 0.026, OR = 0.211) act as strong negative deflators against the senior baseline (>50 years). Overall, are most sensitive to risk salience, experiencing a sharp decline in safety perception when exposed to Television News and Mobile Applications. Overall, place-based offline channels exert a more continuous psychological weight than decentralized digital notifications due to their material embodiment and proximity to daily routines.

**Conclusion:**

The psychological efficacy of crime prevention information is highly context-dependent and spatially bounded. To foster a secure urban environment, city managers must transition from “one-size-fits-all” public broadcasting toward demographically tailored and spatially synchronized communication architectures. Prioritizing reassurance at territorial thresholds while boosting actionable self-efficacy for middle-aged caregiving cohorts can successfully mitigate unintended environmental anxiety and maximize the public health dividends of smart-community governance.

## 1 Introduction

International organizations and local governments are increasingly prioritizing the cultivation of resilient, secure communities (1). Among the myriad factors influencing urban livability and social stability, the fear of crime remains a critical determinant of residents’ subjective well-being, everyday time–space mobility, and their overall quality of life (2–4). When left unaddressed, widespread psychological insecurity can disrupt the social fabric of neighborhoods, restricting vulnerable populations’ access to urban opportunities, and ultimately undermines public health goals (5). To mitigate these concerns, proactive security campaigns, ranging from physical Crime Prevention Through Environmental Design (CPTED) strategies to safety communication initiatives, have been widely deployed globally to deter criminal activity and bolster public confidence (6, 7).

While traditional crime prevention research has long focused on the physical modification of urban landscapes (8, 9), the mechanisms through which a subjective sense of safety is constructed have become increasingly complex. Beyond physical interventions, the dissemination of risk information has emerged as a crucial “soft power” in shaping the public’s psychological landscape (10). Residents no longer assess localized threats solely through the physical presence of environmental disorder. Instead, their safety perceptions are continuously reconstructed through a diverse, hybrid ecosystem of communication channels (11). These channels span a continuum from place-based, physical media anchored within the immediate built environment to decentralized, virtual digital platforms accessible anytime and anywhere (12).

Historically, built environments served as the primary interface for risk communication, utilizing stationary markers such as neighborhood noticeboards, building entrance signs, and elevator lobby posters to foster “territorial reinforcement” (8, 13). These place-based physical cues are designed to manifest official guardianship, theoretically generating environmental reassurance by signaling active community management (14). However, the rapid proliferation of Information and Communication Technologies (ICTs) has superimposed a virtual layer onto this physical grid. Tools such as Mobile Applications, instant messaging groups (e.g., WeChat), and localized SMS alerts offer real-time, high-frequency risk signals that transcend geographical boundaries, creating a form of “virtual guardianship” (15).

Despite the ubiquity of these dual-track information channels, empirical research on their capacity to alter safety perceptions yields conflicting conclusions, a phenomenon conceptualized as the “Information Paradox” in crime prevention (16). While risk advisories are implemented to reassure the public by eliminating uncertainty, they frequently trigger an unintended “warning effect” over the intended “reassurance effect” (13). Crucially, our proposed “Information Paradox” diverges from generic behavioral anomalies like “risk salience” (17) or the infrastructure-led “paradox of security” (18). It conceptualizes a dynamic, channel-mismatched anxiety that is structurally tied to two overlooked dimensions: spatial context and demographic heterogeneity.

On one hand, according to environmental psychology and Prospect–Refuge Theory (19), the psychological decoding of an advisory is heavily contingent upon the architectural layout of the immediate micro-environment where it is encountered (17). This spatial contingency explains why the paradox triggers a “warning effect” predominantly within high-enclosure settings. On the other hand, a resident’s susceptibility to fear is not uniform. While classical criminological frameworks focus on physical frailty as the primary driver of fear, contemporary intersectional perspectives suggest that psychological vulnerability shifts across life stages (20, 21). Middle-aged cohorts, navigating specific familial roles, may exhibit distinct risk sensitivities compared to younger or older generations, which further shapes their interaction with physical or digital risk media (22).

A prominent limitation in the existing literature is that environmental features and media consumption are often examined in isolation, leaving the empirical comparison between place-based physical media and decentralized virtual channels under-theorized. Furthermore, standard evaluations frequently overlook how the micro-spatial configuration of physical delivery (e.g., transitional thresholds versus enclosed spaces) interacts with the demographic realities of the recipients. To bridge these gaps, this study utilizes empirical data from 687 urban residents to systematically examine how exposure to crime prevention information through eight distinct offline and online channels shapes safety perceptions. By integrating spatial enclosure parameters with interaction models across age cohorts, this research clarifies the boundary conditions of risk communication, offering actionable insights for spatially and demographically tailored urban risk governance.

## 2 Literature review

### 2.1 Spatial enclosure, reassurance, and warning effects in offline channels

Traditional community risk communication is fundamentally place-bound, relying on the physical environment to display safety cues. Within the framework of CPTED, physical communication markers, such as building notices, noticeboards, and banners, are not merely administrative tools. They act as symbolic features designed to communicate social control, surveillance, and active management (7, 8). According to Newman (8) Defensible Space Theory, the psychological efficacy of these environmental cues depends significantly on their ability to reinforce territoriality, the clear delineation between public, semi-public, and private zones. When safety tips are positioned at territorial thresholds, such as unit entrances, they function as explicit “signals of guardianship” (14). This boundary reinforcement generates a “reassurance effect” by reassuring residents that the gateway to their private refuge is under active, proactive surveillance, thereby lowering subjective vulnerability (11).

However, environmental criminology also establishes that the psychological decoding of physical information is highly sensitive to spatial variation and micro-environmental context (23). According to Appleton (19) Prospect–Refuge Theory, human safety perceptions are optimized in settings that provide a wide field of vision (prospect) and an empirical sense of enclosure without entrapment (refuge) (17). When risk-laden crime prevention information is introduced into high-enclosure transit pockets, such as elevator lobbies, the theoretical alignment between cue and space fractures. Elevators are architecturally confined, semi-public micro-spaces characterized by limited fields of view and an absence of escape routes, spatial traits that naturally heighten baseline anxiety (19).

Rooted in Skogan (16) social disorganization framework, encountering explicit crime reminders within these vulnerable, captive environments triggers a powerful “Risk Salience” effect. Instead of feeling protected by the advisory, the physical confinement intensifies the resident’s cognitive awareness of threat and immediate susceptibility to victimization, overpowering any reassuring intent. Similarly, heavy-handed or poorly maintained Community Noticeboards and public banners can act as visual signs of physical incivility and disorder (9), leading to alertness fatigue or signaling an uncontained threat within the communal space. Thus, physical preventive cues do not universally enhance safety. Their psychological impact operates as a spatial double-edged sword, heavily mediated by the architectural enclosure of the immediate micro-residential environment.

### 2.2 Online channels and perceived safety

The ongoing integration of Information and Communication Technologies (ICTs) has introduced virtual channels, including instant messaging groups (e.g., WeChat), dedicated Mobile Applications, and SMS broadcasts, into the community safety grid (24). Unlike stationary offline media, digital platforms are characterized by real-time immediacy and decentralized network structures, offering what some scholars term “Informational Empowerment” (10). From the perspective of Routine Activity Theory (25), digital channels can foster “virtual guardianship.” As neighbors share real-time security alerts and mutually coordinate preventive behaviors, they can enhance a community’s perceived collective efficacy and social cohesion, theoretically buffering the fear of crime across spatial boundaries (26).

However, the psychological transition from physical to virtual risk alerts is not seamlessly positive. The Social Amplification of Risk Framework (SARF) provides a robust theoretical foundation for explaining why digital safety channels frequently underperform or backfire (27). SARF posits that as risk information passes through decentralized social stations, it undergoes intensive cognitive amplification or attenuation. Digital channels are uniquely prone to “context collapse” and information overload, where high-frequency, standardized safety alerts lack localized spatial anchors (11).

When a safety warning is delivered via a detached Mobile Application or text message, it strips away the reassuring, visible cues of local management found at physical thresholds like unit doors (23). According to risk perception literature, frequent exposure to abstract, un-anchored digital risk alerts can continuously reinforce the salience of crime, translating specific, distant incidents into an ambient, omnipresent threat (28). Without a tangible physical buffer to signal active environmental control, decentralized digital alerts can amplify subjective anxiety rather than foster public reassurance.

### 2.3 Demographic heterogeneity and perceived safety

The psychological friction generated by the “Information Paradox” does not impact urban populations uniformly; rather, it is heavily moderated by demographic characteristics, particularly across different generations (10, 22). Historically, scholarly attempts to map demographic variations in the fear of crime have relied on the Social Vulnerability Hypothesis (29, 30). This hypothesis posits that a person’s level of fear is a direct function of their physical or social fragility, consistently identifying the older adults and women as the most fearful groups due to their perceived inability to resist physical aggression or recover from victimization (3, 31).

While the social vulnerability framework effectively explains baseline anxieties linked to physical frailty, it exhibits a significant conceptual limitation: it treats vulnerability as a largely static, physical attribute, frequently failing to capture the dynamic socio-economic stresses that evolve across a resident’s life course (20). Emerging research in social psychology and feminist criminology suggests that risk sensitivity is profoundly reshaped by changing household responsibilities and familial roles across distinct life stages (32).

Specifically, as individuals transition into middle age, they often enter the “sandwich generation,” navigating the dual burden of managing careers while safeguarding their households. For these individuals, the perception of community safety extends far beyond personal well-being. It is intrinsically tied to the collective safety of their dependents (22, 33). This deep-seated protective role introduces a critical interaction effect into community risk communication. Middle-aged residents, acting as the primary protective guardians of the household, display heightened cognitive sensitivity to any environmental or digital risk signals that could threaten their family’s security domain (21). When these individuals encounter risk-laden safety reminders, whether through high-enclosure physical channels like elevator lobbies or abstract digital media like Mobile Applications, their deeply embedded protective instincts are highly susceptible to the “warning effect.” Because digital applications communicate risk without providing the reassuring, visible “signals of guardianship” inherent in well-maintained physical boundaries, they are uniquely prone to aggravating psychological insecurity among middle-aged cohorts. Consequently, rather than technological access barriers, the interplay between environmental enclosure, spatial responsibilities, and life-stage caregiving burdens creates a complex psychological matrix that requires rigorous empirical investigation.

## 3 Materials and methods

### 3.1 Data collection

This study uses Nanjing as a case study. According to government statistics, Nanjing achieved a safety satisfaction rate of 99.3% in 2023 and was ranked as ‘China’s Safest City’ for three consecutive years. Crucially, this low-crime environment provides an ideal empirical setting for evaluating risk communication. In a highly secure urban context, residents develop a heightened baseline expectation of safety, meaning that even low-level property offenses can significantly influence their psychological well-being.

Currently, less-severe property crimes, specifically residential burglaries, electric vehicle thefts, and telecom frauds, constitute the vast majority of localized security challenges, accounting for over 75% of total recorded criminal offenses in urban residential neighborhoods. To address these persistent issues, Nanjing’s municipal authorities have established a highly synchronized, dual-track safety communication infrastructure. On one hand, through its comprehensive “Grid Management” (WANG-GE) system, local neighborhood committees maintain a dense network of place-based physical media, deploying property crime advisories across transitional thresholds (unit entrances) and high-enclosure pockets (elevator lobbies). On the other hand, as a pioneering digital “Smart City,” Nanjing boasts near-ubiquitous smartphone penetration, with local authorities continuously broadcasting safety alerts via decentralized virtual channels, including localized SMS networks, official community Mobile Applications, and dedicated WeChat residential groups. Therefore, Nanjing offers a highly suitable multi-channel information matrix where the relative efficacy of place-bound environmental cues and virtual risk channels can be systematically isolated and evaluated.

The empirical data analyzed in this study were derived from an original, primary field survey designed and executed by the research team in November 2022. Titled “Perception and Prevention of Property Crime in Communities in Nanjing,” the primary research purpose of this survey initiative was to systematically investigate how urban residents decode localized security threats and interact with various community crime-prevention information channels. The survey implementation utilized a rigorous, multi-stage stratified random sampling context to ensure metropolitan-level representativeness. First, using neighborhood-level indicators from the Sixth National Population Census of Nanjing (including household registration, age, education, occupation, housing area, building age, monthly rent, tenure type, housing condition, family structure, and marital status), a principal factor analysis was employed to cluster urban neighborhoods into seven distinct socio-demographic categories. To guarantee spatial and social representativeness, the research team selected 40 specific representative communities across Nanjing as formal sampling sites, balancing geographical distribution and the distinctiveness of the factored social districts. The quota of questionnaires allocated to each of the seven community types was determined strictly in proportion to its total resident population.

The questionnaire administration was conducted entirely through face-to-face, in-person interviews via home visits rather than online or mixed channels, maximizing the reliability and depth of responses. Members of the research group visited the designated households within the 40 sampled communities, providing potential participants (adult residents aged 18 and older) with a comprehensive overview of the research aims while guaranteeing absolute data anonymity. To ensure empirical transparency and minimize compliance or social desirability bias, no monetary compensation was provided.

Out of the 788 completed questionnaires collected, 687 were considered valid samples, with invalid responses excluded due to missing critical information (such as personal characteristics and perceptions of safety). To verify the sample’s scope and credibility, we cross-checked the demographic distribution of these 687 respondents against the official benchmark data from the Seventh National Population Census of Nanjing (34). The sample demonstrated a highly consistent and representative fit across gender, age cohorts, education levels, Household Income, and Housing Types (Table 1).

**Table 1:** Comparison of socio-demographic characteristics between the sample and the population of Nanjing.

Variables		Population	Sample
Gender	Males	50.00%	47.80%
	Females	50.40%	52.20%
Age	18–30	9.95%	18.30%
	31–40	17.62%	19.80%
	41–50	17.68%	18.60%
	51–60	15.16%	11.80%
	61 years and over	29.73%	31.45%
Education	Lower secondary and below	29.74%	21.80%
	High school or secondary school	23.43%	24.60%
	Three-year college	16.18%	13.80%
	Undergraduate	23.07%	31.00%
	Postgraduate and above	7.56%	8.70%
Annual Household Income	Under 80,000	44.00%	39.50%
	80,000–150,000	31.00%	32.80%
	150,000–300,000	20.00%	19.20%
	More than 300,000	6.00%	8.50%
Housing Type	Homeowner	78.20%	82.50%
	Renter	21.80%	17.50%

The core explanatory variables in this study refer to the multi-channel dissemination of crime prevention information. To capture the diversity of information touchpoints, we categorized these channels into Physical (Offline) and Virtual (Online/Broadcast) platforms.

### 3.2 Dependent variable

Perceptions of safety reflect residents’ subjective concerns regarding crime and were assessed using a 5-point Likert scale (Table 2). Specifically, respondents were asked: ‘How safe do you feel living in this neighborhood?’ Response options ranged from 1 (‘very unsafe’) to 5 (‘very safe’). Descriptive analysis reveals that the sampled residents reported a moderate-to-high level of safety, with an average score of 3.40 and a standard deviation of 0.79.

**Table 2:** Descriptive statistics of the dependent variable.

Dependent variable	Variable assignment	Average value	Standard deviation
Residents’ perception of safety	Very unsafe = 1, unsafe = 2, neutral = 3, safe = 4, very safe = 5	3.40	0.79

### 3.3 Explanatory variables

This study categorized crime prevention channels into eight types: Community Noticeboards, unit entrances, elevator lobbies, banners, corridors, text messages, Television News, and mobile apps. The survey included the following question: ‘Where do you typically encounter property crime prevention reminders within your community?.’ Respondents were asked to identify the specific locations or media through which they had received property crime prevention advisories within the past 6 months. If a respondent selected any option, they were considered to have received crime prevention tips through that channel, and the corresponding option was recorded as a value of 1. Conversely, if no option was selected, the value was recorded as 0.

From the theoretical standpoint of environmental psychology and communication research, a critical conceptual distinction must be made between information exposure (the spatial encounter with a cue) and information perception (the internal cognitive processing and subjective appraisal of that cue). While measuring subjective levels of trust or cognitive processing for each channel would offer psychological depth, we deliberately adopted an objective, behavioral exposure framework (0/1 binary coding) for rigorous methodological reasons. In cross-sectional survey research, utilizing highly subjective psychological metrics to evaluate both the independent variables (media appraisal) and the dependent variable (safety perception) can introduce severe endogeneity bias and common method variance (CMV). By anchoring our explanatory variables to the objective behavioral fact of environmental exposure, we successfully decouple the spatial stimulus from the psychological outcome, thereby isolating the net directional impact of different communication infrastructures on public safety perceptions.

Residents’ perception of safety cues is measured by an additive index based on the following eight channels. For the Physical Channels, variables include Community Noticeboards, Unit Entrances, Elevator Lobbies, Banners, and Corridors. Each was treated as a binary variable (1 = Perceived; 0 = Not Perceived). These channels represent localized, place-based cues that signal communal guardianship.

For the Virtual/Broadcast Channels, the study included Text Messages (SMS), Mobile Applications, and Television News. Similarly, these were coded as binary variables. In the subsequent ordinal regression models, these channels were entered both as main effects and as interaction terms with age cohorts to test the potential “Reassurance vs. Warning” effects.

### 3.4 Control variables

The selection of control variables is grounded in the Social Vulnerability Hypothesis, which posits that individuals with limited resources perceive themselves as more susceptible to victimization (35). Gender is included as a binary control (0 = Male; 1 = Female), under the assumption that females typically report higher fear of crime due to perceived physical vulnerability (36). Education level is measured on a 5-point scale (ranging from 1 = “Less than Junior School” to 5 = “Postgraduate Degree”), and Annual Household Income is categorized into six incremental levels (e.g., from 1 = “Under 30,000 RMB” to 6 = “Over 1,000,000 RMB”). These factors serve as proxies for socio-economic status, as higher-income and more educated individuals may possess greater resources to mitigate perceived risks (37). Age is treated as a critical moderating variable and is operationalized as a categorical factor with six cohorts (1 = 18–30; 2 = 31–40; 3 = 41–50; 4 = 51–60; 5 = Over 60). This allows the model to capture life-cycle differences in technology affinity and risk sensitivity (38).

Beyond individual traits, residential and environmental factors are controlled to account for the impact of one’s immediate living conditions. We included Housing Type as a binary indicator of housing tenure (1 = Homeowner; 2 = Renter). According to the Social Organization Theory, homeowners often exhibit higher levels of neighborhood attachment and are more likely to participate in local surveillance, which can lead to higher baseline safety perceptions compared to renters (39).

### 3.5 Analytic strategy

Since the dependent variable, residents’ perception of safety, is measured on a 5-point Likert scale representing a natural rank order, this study employs ordinal logistic regression (OLR) models. All statistical data analysis and modeling procedures were implemented using IBM SPSS Statistics (Version 24.0). To test the hypothesized moderating role of demographic characteristics, the analysis proceeds in stages. Beyond assessing the main effects of information channels, interaction terms between age cohorts and specific delivery methods (e.g., Age and Mobile App) are introduced into the models. This allows us to examine whether the effectiveness of a particular communication strategy, or its potential “warning effect,” varies across different segments of the population.

To ensure structural validity, the score test for the proportional odds (parallel lines) assumption and Pearson goodness-of-fit tests were systematically conducted for both the baseline main-effects model and the subsequent interaction model, with the comprehensive diagnostic outcomes tabulated and discussed in Section 4.2 (Table 3). Therefore, the standard OLR specification was retained as the most parsimonious and theoretically interpretable approach for testing our hypotheses.

**Table 3:** Ordinal logistic regression estimates of multi-channel crime prevention tips on residents’ safety perceptions (*N* = 687).

Variable and levels	Coefficient (*B*)	Std. error	Wald	Sig. (*p*)	Odds ratio (OR)	95% CI for OR [LL, UL]
Thresholds						
Safety perception = 1	−5.042	2.947	2.927	0.087	–	–
Safety perception = 2	−3.288	2.938	1.252	0.263	–	–
Safety perception = 3	−1.028	2.937	0.122	0.726	–	–
Safety perception = 4	1.632	2.936	0.309	0.578	–	–
Control variables						
Gender male	0.328	0.147	4.934	0.026*	1.388	[1.040, 1.853]
Gender female (Ref)	0	–	–	–		
Age [V2 = 1]	−1.897	1.987	0.911	0.34	0.150	[0.003, 7.374]
Age [V2 = 2]	−2.091	1.987	1.108	0.293	0.124	[0.003, 6.068]
Age [V2 = 3]	−2.485	1.983	1.569	0.21	0.083	[0.002, 4.057]
Age [V2 = 4]	−2.144	1.984	1.168	0.28	0.117	[0.002, 5.720]
Age [V2 = 5]	−1.887	1.971	0.916	0.338	0.152	[0.003, 7.214]
Age [V2 = 6] (Ref)	0	–	–	–	–	–
Education [V3 = 1]	−0.556	0.347	2.572	0.109	0.574	[0.290, 1.132]
Education [V3 = 2]	−0.570	0.329	3.003	0.083	0.566	[0.296, 1.078]
Education [V3 = 3]	−0.341	0.291	1.161	0.281	0.711	[0.366, 1.380]
Education [V3 = 4]	−0.313	0.291	1.161	0.281	0.731	[0.414, 1.292]
Education [V3 = 5] (Ref)	0	–	–	–	–	–
Household Income [V4 = 1]	1.615	1.928	0.701	0.402	5.028	[0.115, 220.081]
Household Income [V4 = 2]	1.589	1.921	0.685	0.408	4.899	[0.114, 211.451]
Household Income [V4 = 3]	1.270	1.918	0.439	0.508	3.561	[0.083, 152.929]
Household Income [V4 = 4]	1.318	1.923	0.470	0.493	3.736	[0.086, 162.062]
Household Income [V4 = 5]	1.203	1.940	0.384	0.535	3.330	[0.074, 149.302]
Household Income [V4 = 6] (Ref)	0	–	–	–	–	–
Housing Type [V5 = 1]	−0.22	0.209	1.104	0.293		
Housing Type [V5 = 2] (Ref)	0	–	–	–	–	–
Offline channels						
Community Noticeboards	−0.408	0.171	5.702	0.017*	0.665	[0.476, 0.930]
Unit entrance	0.37	0.176	4.437	0.035*	1.448	[1.026, 2.042]
Elevator lobbies	−0.741	0.267	7.701	0.006**	0.477	[0.282, 0.804]
Banner	0.252	0.259	0.945	0.331	1.287	[0.774, 2.136]
Corridor	0.152	0.216	0.494	0.482	1.164	[0.763, 1.777]
Online channels						
Text message	0.016	0.17	0.009	0.925	1.016	[0.728, 1.419]
Television News	−0.068	0.237	0.082	0.775	0.934	[0.587, 1.487]
Mobile Application (App)	−0.364	0.237	2.372	0.124	0.695	[0.437, 1.104]
Model diagnostic indices						
Model fitting likelihood ratio $\chi^{2}$	57.676			0.001		
Goodness-of-fit (Pearson $\chi^{2}$ )	2290.444			0.286		
Goodness-of-fit (deviance $\chi^{2}$ )	1398.418			1.000		
Test of parallel lines $\chi^{2}$	102.275			0.004		
Cox and Snell pseudo *R*^2^/Nagelkerke *R*^2^	0.081	/	0.088			

LL, Lower Limit; UL, Upper Limit. The Odds Ratios (OR) and 95% Confidence Intervals are exponentiated parameters (eB). (Ref) indicates the reference category for the respective variable group. *: *p* < 0.05, **: *p* < 0.01.

Regarding data integrity, missing values were addressed using Multiple Imputation (MI) to prevent systematic bias and maintain statistical power. Across the *N* = 687 dataset, the missingness was exceptionally minimal: baseline predictors including gender, age, education, and all informational channels were completely intact (0% missingness), while Housing Type and Household Income exhibited minor missingness of only 15 cases (2.18%) and 10 cases (1.46%), respectively. Assuming a Missing-at-Random (MAR) mechanism, we performed MI via the Fully Conditional Specification (FCS) algorithm in SPSS, generating *m* = 5 distinct imputed datasets. Categorical constraints were strictly maintained during the iterative process to avoid floating-point errors. By pooling the estimates from these 5 complete matrices using Rubin’s (40) rules, we ensured that the final ordinal logistic regression parameters are robust, unbiased, and representative of the sampled population.

## 4 Results

### 4.1 Descriptive statistics and sample profile

Descriptive statistics (Table 1) provide an overview of the respondents’ characteristics. The gender distribution was relatively balanced, with males and females accounting for 47.80 and 52.20% of the sample, respectively. Notably, the sample includes a significant proportion of older adults residents (31.45% aged 61 or older), reflecting the aging demographic of central Nanjing. Economically, the largest segment of respondents (39.50%) reported an Annual Household Income below 80,000 RMB, and a substantial majority (82.50%) were homeowners, indicating a stable residential population.

Regarding the perception of crime prevention information channels (Table 4), a diverse landscape of information accessibility emerges. Both Community Noticeboards (37.0%) and text messages (37.0%) were identified as the most prevalent sources, indicating a dual-reliance on traditional physical interfaces and direct digital alerts. Among other offline channels, unit entrances (26.0%) and corridors (15.0%) remained significant touchpoints for residents, whereas the elevator lobby (9.0%) and banners (9.0%) were perceived with considerably lower frequency. In the digital and broadcast realm, Television News and Mobile Applications were each reported by 13.0% of respondents. These patterns suggest that while decentralized digital tools are proliferating, localized physical cues in the immediate residential environment, specifically noticeboards and entrances, continue to play a foundational role in the dissemination of safety advisories.

**Table 4:** Descriptive statistics for the explanatory variable.

Variable		Meaning and assignment of variables	Frequency (*N* = 1)	Percentage (%)	Mean	SD	VIF
Offline	Community Noticeboards	Yes = 1, No = 0	254	37.0%	0.37	0.48	1.00
	Unit entrance	Yes = 1, No = 0	179	26.1%	0.26	0.44	1.15
	Elevator lobby	Yes = 1, No = 0	62	9.0%	0.09	0.29	1.11
	Banner	Yes = 1, No = 0	62	9.0%	0.09	0.29	1.04
	Corridor	Yes = 1, No = 0	103	15.0%	0.15	0.35	1.07
Online	Text message	Yes = 1, No = 0	254	37.0%	0.37	0.48	1.11
	Television News	Yes = 1, No = 0	89	13.0%	0.13	0.34	1.05
	Mobile App	Yes = 1, No = 0	89	13.0%	0.13	0.34	1.12

The multicollinearity of several variables was assessed by analyzing their Tolerances and VIF (Table 4). Eight variables, including Community Noticeboards, unit entrance, elevator lobby, banner, corridor, text message, Television News, and mobile app, had VIF values ranged from 1 to 1.15. The VIF results indicate that the multicollinearity among these variables is not significant. The variables are relatively independent and suitable for use in multivariate logistic regression. This provides a solid data foundation for subsequent analysis, ensuring the stability and explanatory power of the model.

### 4.2 Main effects of information channels and controls

Table 3 illustrates the relationship between residents’ perceptions of crime prevention tips and their sense of safety, as indicated by their statistical significance in the ordinal logistic regression. The results reveal that several variables significantly influence the enhancement of safety perception, providing a quantitative foundation for optimizing the design of safety prompts.

The score test for the proportional odds (parallel lines) was conducted (Table 3). In the baseline main-effects model, the test yielded a chi-square value of 102.275 (df = 67, *p* = 0.004), and in the subsequent interaction model, the chi-square was 70.746 (df = 40, *p* = 0.02). Although these *p*-values are less than 0.05, prior methodological literature widely recognizes that the parallel lines test is notoriously hypersensitive to large sample sizes (*N* = 687 in this study), frequently rejecting the null hypothesis even when the model parameters remain highly stable and substantively parallel. Crucially, our models demonstrated exceptional global adequacy according to the Pearson goodness-of-fit tests, which are highly stable indicators of model specification. For the main-effects model, the Pearson chi-square was 2290.444 (df = 2,213, *p* = 0.286 > 0.05), and for the interaction model, it was 2273.189 (df = 2,240, *p* = 0.307 > 0.05). The fact that both goodness-of-fit *p*-values are substantially greater than 0.05 strongly proves that the data successfully fit the proportional odds OLR framework. The models also achieved satisfactory Cox and Snell pseudo-*R*^2^ metrics (0.081 for the main model and 0.098 for the interaction model).

Among the physical (offline) channels, three specific locations significantly influence safety perceptions, showing a mix of “reassurance” and “warning” effects. Specifically, Unit Entrances were found to have a significant positive impact on residents’ sense of security (*B* = 0.370, *p* = 0.035). This suggests that when residents notice crime prevention advisories at the entrance of their building, it functions as a signal of formal guardianship, thereby enhancing their perceived safety.

Conversely, Community Noticeboards and Elevator Lobbies both demonstrate significant negative associations with safety perceptions. Exposure to information via Community Noticeboards significantly reduces the likelihood of feeling safe (*B* = −0.408, *p* = 0.017). A more pronounced negative effect was observed for elevator lobbies, which yielded the strongest significant coefficient in the model (*B* = −0.741, *p* = 0.006). These findings indicate that in these specific semi-public spaces, preventive reminders may inadvertently heighten the salience of crime risks, triggering a “warning effect” that diminishes psychological security. Other physical markers, such as Banners (*B* = 0.252, *p* = 0.331) and Corridors (*B* = 0.152, *p* = 0.482), did not reach statistical significance.

Regarding virtual and broadcast channels, none of the digital platforms, including Text Messages (*B* = 0.016, *p* = 0.925), Television News (*B* = −0.068, *p* = 0.775), and Mobile Applications (*B* = −0.364, *p* = 0.124), showed a statistically significant direct impact on the overall sample’s perception of safety. This lack of significance in the main effects model suggests that the influence of digital risk communication may be more nuanced, potentially being moderated by individual characteristics such as age, or that these channels are perceived with less immediacy compared to localized physical cues in the residential environment.

Among the socio-demographic factors, gender emerged as a statistically significant predictor of perceived safety (*B* = 0.328, *p* = 0.026). The positive coefficient for males indicates that, consistent with the vulnerability hypothesis, male residents report significantly higher levels of perceived safety than their female counterparts.

In contrast, other demographic variables, including age cohorts, education level, Household Income, and housing tenure, did not reach statistical significance in this model. Specifically, although the coefficients for all age groups were negative, suggesting a potential downward trend in safety perception compared to the reference group, these differences were not statistically robust (*p* > 0.05). Similarly, neither the lowest education level (*B* = −0.556, *p* = 0.109) nor the lower income bracket (*B* = 1.615, *p* = 0.402) showed a significant direct association with safety scores. The lack of significance for housing tenure (*B* = −0.220, *p* = 0.293) suggests that in this specific urban context, the distinction between homeowners and renters does not fundamentally alter the baseline perception of security when other environmental and informational factors are held constant. These results highlight that gender remains the most stable demographic determinant of fear of crime in the sampled population.

### 4.3 Interaction analysis between age and communication channels

To further explore how the impact of crime prevention information varies across different life stages, interaction terms between age cohorts and communication channels were introduced into the model (Table 5). The results reveal significant heterogeneous effects, particularly for traditional media and specific digital platforms. Importantly, the reported Odds Ratios (OR) derived from these interaction terms represent multiplicative adjustments relative to the designated reference baseline (residents aged>50 years who did not utilize the respective channel) and their corresponding main effects, rather than direct subgroup effects in isolation.

**Table 5:** Summary of interaction effects between age cohorts and communication channels on perceived safety.

Interaction term (age × channel)	Coefficient (*B*)	Std. error	Wald	Sig. (*p*)	Odds ratio (OR)	95% CI for OR [LL, UL]
Offline channels						
(18–30 years) × Television News	−1.324	0.676	3.829	0.050	0.266	[0.071, 1.002]
(31–40 years) × Television News	−1.985	0.645	9.468	0.002	0.137	[0.039, 0.486]
(41–50 years) × Television News	−1.324	0.682	3.767	0.052	0.266	[0.070, 1.013]
(41–50 years) × Community Noticeboards	−0.830	0.460	3.255	0.071	0.357	[0.177, 1.075]
Online channels						
(41–50 years) × Mobile Application	−1.556	0.701	4.927	0.026	0.211	[0.053, 0.834]

LL, Lower Limit; UL, Upper Limit. The Odds Ratios (OR) and 95% Confidence Intervals are exponentiated parameters (eB), representing the multiplicative change in the odds of higher safety perceptions relative to the reference groups (age >50 years and respective channel baseline).

The most striking and statistically robust interaction is observed between Television News (broadcast channel) and the early-middle-aged cohort. Specifically, the interaction term (31–40 years) × Television News is negative and highly significant [*B* = −1.985, *p* = 0.002, OR = 0.137, 95% CI: (0.039, 0.486)]. This indicates that, holding other factors constant, the association between television news consumption and higher safety perceptions is systematically and heavily attenuated among residents aged 31–40 relative to the senior reference group (aged greater than 50). Crucially, in alignment with methodological caution, several other interaction parameters approached but did not surpass the conventional significance threshold (*α* = 0.05) and must be strictly treated as marginally significant or trend-level findings rather than robust configurations. Specifically, the interaction of (18–30 years) × Television News [*B* = −1.324, *p* = 0.050, OR = 0.266, 95% CI: (0.071, 1.002)] sits precisely on the statistical boundary, while the interaction of (41–50 years) × Television News [*B* = −1.324, *p* = 0.052, OR = 0.266, 95% CI: (0.070, 1.013)] and the physical channel interaction of (41–50 years) × Community Noticeboards [*B* = −0.830, *p* = 0.071, OR = 0.357, 95% CI: (0.177, 1.075)] exhibit marginal, near-threshold tendencies. These parameters are explicitly isolated from our definitive conclusions and interpreted merely as potential empirical trends, suggesting that risk sensitivity across broadcast and physical notification networks might fluctuate depending on life stages.

In the fully significant digital domain, a robust negative interaction emerged between Mobile Applications and the intermediate age cohort. The interaction term (41–50 years) × Mobile Application is statistically significant [*B* = −1.556, *p* = 0.026, OR = 0.211, 95% CI: (0.053, 0.834)]. Rather than implying a direct risk reduction within this cohort in isolation, this finding demonstrates that the likelihood of reporting higher safety perceptions when receiving information via mobile apps is significantly lower for middle-aged residents than for the older baseline cohort. This indicates that the relationship between digital channel usage and public safety evaluations is highly heterogeneous across life stages, with middle-aged cohorts displaying a significantly less receptive response pattern compared to their older counterparts.

To fully unlock the interpretability of this moderation framework, these interaction effects must be synthesized with their corresponding main effects (Table 3). In our model specification, the main effects of the communication channels capture the baseline relationship for the reference age group (residents aged greater than 50 years), while the main effects of the age cohorts capture the safety perception differentials among residents who do not utilize those specific channels. For instance, the main effect of Television News represents the baseline impact on the oldest cohort (aged greater than 50), which typically reflects a stabilizing or non-negative baseline association with safety evaluations. However, when examining the younger cohorts, the significant negative interaction terms, such as (31–40 years) × Television News, act as multiplicative deflators against this baseline, indicating that the reassuring relationship observed among older adults residents is inverted or neutralized for younger demographics. Similarly, regarding digital pathways, the significant negative interaction of (41–50 years) × Mobile Application reveals a steep divergence from the senior benchmark, demonstrating that the intermediate age cohort experiences a significantly lower likelihood of high safety alignment via digital alerts. Synthesizing these parameters clarifies that communication channels do not affect public safety perceptions uniformly. Rather, their efficacy is strictly contingent upon the recipient’s life stage, with younger and middle-aged groups exhibiting a systematically higher sensitivity to risk discourse in both traditional broadcast and smart digital environments.

## 5 Discussion

This paper examines the relationship between perceived crime prevention tips, delivery channels, and residential safety perceptions among a sample of residents in Nanjing, China. By evaluating both physical environmental cues and modern digital notifications across different generational cohorts, the findings offer new insights into how public safety communications modulate urban livability.

### 5.1 Refined defensible space: spatial enclosure and the Information Paradox

This study highlights that the statistical association between physical crime prevention tips and safety perceptions is highly polarized based on micro-spatial placement, uncovering a distinct “Information Paradox” in residential communities. Specifically, encountering safety advisories at unit entrances significantly enhances residents’ safety perceptions, whereas experiencing them inside elevator lobbies corresponds to a notable decrease in security feelings.

This spatial variation refines Newman (8) Defensible Space Theory and Appleton (19) Prospect–Refuge Theory, while actively extending recent empirically grounded localized safety literature [e.g., Navarrete-Hernandez et al. (1); Troy et al., (41)]. Our findings demonstrate that physical preventive cues do not universally foster reassurance. Their efficacy depends heavily on the degree of spatial enclosure and territorial definition. Unit entrances function as crucial “territorial thresholds” separating public communal tracks from private domains. In alignment with modern extensions of territoriality (8), placing tips at this threshold reinforces visible signs of formal control and active building management, thereby functioning as a positive predictor of safety.

Conversely, the negative association observed in elevator lobbies uncovers a critical micro-space constraint where structural entrapment overrides message intent. Elevators are inherently confined, high-enclosure transit zones characterized by a restricted field of view and zero escape routes. Recent visual and environmental studies in urban settings [e.g., Gargiulo and Garcia (21); Kamani Fard and Paydar (42)] confirm that enclosed, low-refuge transit pockets inherently exacerbate vulnerability. These spatial features are identified as major catalysts for fear according to Prospect–Refuge theory (17, 19). When explicit crime reminders are introduced into these captive spaces, they appear to function as situational cues of vulnerability, shifting residents’ focus toward immediate environmental risks. This aligns with contemporary findings by Chen et al. (43), who noted that safety information delivered in poorly designed or highly enclosed urban micro-spaces frequently backfires, amplifying a negative relationship with perceived security.

### 5.2 Comparison of online and offline channels

A major observation of this study is the sharp discrepancy between information channels: offline environmental tips exert a far more substantial and continuous influence on safety perceptions than digital communication channels (such as mobile apps or WeChat groups), which generally failed to show significant main effects. This variance suggests that place-based physical cues possess a unique psychological weight that decentralized digital platforms cannot replicate, enriching the ongoing discourse on environmental criminology in the digital era.

This discrepancy can be explained through the lens of spatial embodiment and Cohen and Felson (25) Routine Activity Theory, contrasting with recent claims regarding the total digitization of urban governance [e.g., van der Sluis (44)]. Offline tips are physically embedded within the material landscape and woven into the structured spatial routines of residents’ daily lives, creating a continuous and visible presence that reinforces memory (1). More importantly, as Jiang et al. (24) propose, residents do not just process the direct text. They use tangible environmental cues to predict the future social uses and human activities of a given space. A localized notice physically anchored to a community wall or entrance is decoded as an authoritative sign of active, bottom-up guardianship, encouraging residents to predict an increase in desirable routine activity and formal control.

In contrast, digital media lacks this spatial specificity and physical embodiment. Standardized, mass-broadcasted app notifications suffer from extreme context collapse (11). As highlighted in recent digital communication studies (45), high-frequency digital safety alerts often suffer from information overload, causing users to dismiss them as generic background noise. Consequently, they fail to meaningfully alter the subjective assessment of immediate, localized physical risks (11).

This pervasive non-significance of digital vectors in our main-effects model carries profound theoretical implications, as it directly challenges the conventional Social Amplification of Risk Framework (SARF) (27). SARF typically posits that modern mass media and decentralized communication channels act as powerful institutional and social pipelines that systematically amplify public risk salience and heighten fear of crime (46). Our empirical null effects suggest a critical structural boundary to this framework within contemporary smart-governance landscapes, a phenomenon we conceptualize as “digital desensitization” through spatial detachment. In the saturated informational ecology of smart cities, high-frequency, non-place-based digital alerts often trigger a severe “information overloading” effect, causing individuals to develop psychological immunity or coping avoidance strategies (47). Because these digital streams lack geographic friction and concrete material presence, they are easily decoupled from the resident’s immediate physical reality (11). Consequently, rather than amplifying risk, these placeless informational vectors are filtered out as chronic cognitive static. SARF, therefore, does not hold universally; its capacity to amplify or attenuate risk perceptions is strictly bounded by the media’s spatial embodiment and its proximity to the localized routines of daily life.

### 5.3 Life stages and intersectional vulnerability

When examining demographic differences, our interaction analysis yields a counter-intuitive finding that challenges the traditional Social Vulnerability Hypothesis, which typically posits the older adults as the most sensitive to safety threats. Instead, our results demonstrate that younger-to-middle-aged residents (aged 31–40 and 41–50) exhibit a significantly different response pattern when exposed to traditional media and Mobile Applications. Conversely, the senior cohort (aged >50, including older adults residents over 60) displays no distinct or significant interaction fluctuations, maintaining a stable baseline alignment with communication channels.

This unexpected variance extends traditional fear of crime literature by proving that risk sensitivity across life stages is driven not just by physical weakness (20), but by socio-economic roles and spatial responsibilities. This empirical pattern strongly shifts the explanatory mechanism away from technology-driven frameworks, since the older baseline cohort exhibits no digital-access rejection, and firmly relocates it within a life-course structural vulnerability model. This finding directly corroborates recent empirical work by Hirtenlehner and Farrall (48), who argued that fear of crime in contemporary cities is deeply structural, tied to caregiving duties and social investment rather than age-related physical limitations alone. Younger and middle-aged cohorts frequently serve as the primary guardians and economic providers for household dependents (minor children and aging parents).

When these cohorts encounter crime reminders via mobile apps, the association with high safety evaluations is significantly attenuated compared to the older reference baseline (>50 years). Because digital platforms lack the reassuring, tangible attributes and visible “signals of guardianship” found at physical thresholds (like unit entrances), they offer no situational buffer. This structural divergence resonates with recent smart-city critiques [e.g., Kummitha et al. (49)], which suggest that continuous digital alerts can create an unbuffered exposure to risk discourse, resulting in a less receptive response pattern among individuals heavily invested in familial protection.

### 5.4 Practical implications

The practical value of this research lies in its empirical critique of “one-size-fits-all” public safety broadcasting, providing a clear blueprint for urban policymakers to design safer, more equitable residential environments (50). To maximize public psychological well-being, safe public spaces, city managers must transition toward a spatially and demographically tailored risk communication strategy.

Authorities should strategically allocate resources based on the spatial concentration of fear (23). Reassuring safety tips should be prioritized at territorial thresholds like unit entrances to reinforce signals of informal guardianship (8). Conversely, managers must exercise extreme caution in high-enclosure transit pockets like elevator lobbies, omitting detailed crime reports to prevent activating spatial vulnerability and entrapment anxiety (19).

Given that younger and middle-aged cohorts shoulder heavy caregiving responsibilities for their household dependents, risk communication directed at this age group must shift from merely raising risk awareness to boosting actionable self-efficacy (20, 22). Furthermore, digital smart-city platforms should move away from high-frequency, standardized text blasts that trigger alertness fatigue (16). Instead, digital alerts should provide localized, neighborhood-level updates that encourage residents to predict positive, routine social control within their immediate environments (11, 24).

### 5.5 Limitations

Despite its empirical and theoretical insights, several specific limitations of this study should be acknowledged. First, while the use of single-item indicators to measure subjective safety perception aligns with broad-scale municipal security evaluations and successfully minimized respondent fatigue during our intensive face-to-face home visits, it poses inherent constraints regarding measurement reliability and construct validity. In quantitative behavioral analysis, single-item metrics can be more susceptible to random measurement error compared to multi-item latent scales. When utilizing ordinal logistic regression and complex demographic interaction models, this measurement constraint may restrict statistical power or obscure more nuanced psychological variations.

Additionally, our binary measurement of communication channels captures immediate information exposure rather than source credibility or the psychological depth of perception. Crucially, as our structured questionnaire was designed for a broad empirical census, we did not directly measure individual psychometric traits or underlying cognitive mediating mechanisms (such as generalized anxiety or specific risk sensitivity). Consequently, our findings support robust statistical associations and heterogeneous response patterns across age cohorts, but cannot definitively map the underlying psychological pathways. Future research should deploy multi-item psychometric scales (e.g., separating affective fear, cognitive risk perception, and behavioral constraints) to further strengthen the explanatory power of these multi-channel dynamics.

Second, the cross-sectional architecture of this research limits our capacity to definitively establish causal trajectories, introducing the possibility of reverse causality. For instance, residents with a baseline of lower safety perceptions or higher generalized anxiety may exhibit selective attention, actively seeking out or noticing physical and digital crime prevention cues more frequently than their reassured counterparts. To resolve this endogeneity, future designs should deploy longitudinal panel surveys or experimental manipulations of message framing to isolate true causal vectors. Furthermore, our geographical focus on a single major metropolis raises distinct generalizability concerns. Nanjing is characterized by an exceptionally high public safety satisfaction rate (e.g., reaching 99.3% in official 2023 censuses). This localized institutional context creates a pronounced “ceiling effect” that heavily suppresses variance in the dependent variable, potentially truncating or masking the statistical main effects of decentralized digital channels.

To expand the boundary conditions of the spatial-demographic interaction model established here, subsequent investigations should pursue multi-city comparative designs that span cities with divergent crime rates and baseline safety levels. Finally, future work should strive to incorporate objective neighborhood-level crime data (e.g., geocoded police dispatch records) alongside subjective surveys. Controlling for objective environmental risk will allow GeoAI and urban communication researchers to more precisely disentangle the psychological reassuring effects of localized messaging from the structural realities of urban crime geometries.

## 6 Conclusion

This study offers a nuanced empirical examination of how crime prevention communication modules influence residential safety perceptions within contemporary urban environments. By synthesizing environmental criminology with lifecourse perspectives, our findings yield three core conclusions:

First, our results validate the existence of a distinct “Information Paradox” in public safety communication, demonstrating that the psychological impact of preventive messaging is strictly bounded by the spatial micro-environment. Physical cues deployed at unit entrances function as territorial thresholds that significantly enhance safety perceptions by projecting visible signals of active management and formal guardianship. Conversely, identical reminders introduced into highly enclosed or transitional spaces, such as elevator lobbies, trigger a counter-productive “warning effect.” In these captive settings, structural entrapment and low-refuge geometry override the message’s reassuring intent, inadvertently amplifying immediate environmental risk salience and diminishing residents’ subjective security.

Second, this research uncovers a sharp structural discrepancy between offline physical cues and online digital channels. Place-based offline communication exerts a far more substantial and continuous influence on safety evaluations due to its spatial embodiment within the material landscape and its seamless integration into residents’ structured daily routines. In contrast, decentralized digital vectors suffer from severe context collapse and information overloading. High-frequency, placeless smart-city alerts often trigger a “digital desensitization” effect, causing individuals to filter out digital notifications as chronic cognitive static, thereby failing to alter subjective assessments of localized physical risks.

Third, life-course demographics act as a pivotal moderator of risk perception, shifting the explanatory focus away from technology-access frameworks toward socio-economic roles and spatial responsibilities. Our interaction analysis reveals that younger-to-middle-aged residents (specifically the 31–40 and 41–50 age cohorts) exhibit a systematically higher sensitivity to risk discourse across both traditional broadcast media and smart digital networks compared to the older baseline population. This heightened receptivity is driven by their structural roles as primary household guardians and economic providers. Heavily burdened with caregiving duties for both minor children and aging parents, these intermediate cohorts experience a significant inversion or attenuation of reassurance when exposed to unbuffered risk information.

In terms of practical community governance, these insights demand an immediate paradigm shift from “one-size-fits-all” public broadcasting toward a Targeted and Spatially Synchronized Communication Strategy. Urban managers and municipal authorities should strategically allocate communication resources: prioritizing positive, reassuring signals at structural territorial entryways, while exercising extreme caution in high-enclosure transit pockets. Furthermore, digital smart-governance platforms must move away from standardized text blasts, pivoting instead toward localized, action-oriented messaging that boosts self-efficacy for risk-sensitive caregiver demographics. By calibrating information delivery with the distinct spatial and demographic realities of urban neighborhoods, city authorities can successfully mitigate environmental anxiety and maximize the public health dividends of smart-community initiatives.

## Data Availability

The raw data supporting the conclusions of this article will be made available by the authors, without undue reservation.
